# Quality by design based ecofriendly HPLC analytical method for simultaneous quantification of erastin and lenalidomide in mesoporous silica nanoparticles

**DOI:** 10.1038/s41598-025-93331-8

**Published:** 2025-03-14

**Authors:** Ashutosh Gupta, S. P. Rachana, Sudheer Moorkoth, Namdev Dhas

**Affiliations:** 1 Department of Pharmaceutical Quality Assurance, Manipal College of Pharmaceutical Sciences, Manipal Academy of Higher Education, Manipal, 576104 Karnataka India; 2 Department of Pharmaceutics, Manipal College of Pharmaceutical Sciences, Manipal Academy of Higher Education, Manipal, 576104 Karnataka India

**Keywords:** Box-Behnken design, Mesoporous silica nanoparticles, HPLC, Design of expert, Nanoformulation, Cancer, Health care, Oncology, Nanoscience and technology

## Abstract

The aims of this work to optimize and validate a RP-HPLC method to quantify erastin (ERT) and lenalidomide (LND) in mesoporous silica nanoparticles (MSNs). The Design of Experiments (DoE) strategy optimized the RP-HPLC method. The independent variables were buffer ratio, buffer pH, flow rate and injection volume. The dependent variables were retention time (Rt), Peak area, and resolution between the peaks of the analytes. The optimized conditions were: buffer ratio 68% and methanol 32%, flow rate 0.8 mL/min, buffer pH 5.8, and injection volume 10 µL. The ICH Q2(R1) recommendations were followed in the validation of the optimized RP-HPLC method. The method demonstrated linearity of more than 0.99 for both ERT and LND. The LOD and LOQ were 0.75 and 1.62 ng/mL for ERT; for LND 31.25 and 50 ng/mL. The specificity of the established RP-HPLC method was unaffected by the MSNs matrix. The drugs-loaded MSNs were analyzed using the suggested RP-HPLC technique. The % entrapment efficiency of ERT and LND was found to be 72.65 and 79.50%, and drug loading of ERT and LND was found to be 14 and 17% in MSNs, respectively. The optimized RP-HPLC method was used to check the in-vitro drug release of the ERT and LND from the ERT-LND@MSNs. Surface properties of synthesized MSNs was checked through particle and SEM analysis. The developed analytical method was eco-friendly according to AGREE analysis and GAPI analysis.

**Supplementary Information:**

The online version contains supplementary material available at 10.1038/s41598-025-93331-8.

## Introduction

Tumor is the leading reason of death globally. Gliomas include around 30% of all glioblastoma, including 80% of malignant ones. In the past, the World Health Organization (WHO) divided gliomas into four classes according to their histological features: grade IV (glioblastoma), grade III (anaplastic gliomas), and grade I and II (low-grade gliomas)^1^, which stand for different cancer stages. In recent years, a notable advancement in the development of novel strategy for categorizing and treating gliomas is primarily due to the introduction of genomic, transcriptome, and epigenetic profiling. These ideas will support the morphology-only categorization scheme. GBM is the most prevalent and malignant kind of primary brain tumor, making up as much as 50% of all gliomas. Despite improvements in the present standard of care, which consists of radiation, surgery, and medicine (often chemotherapy combined with concurrent temozolomide (TMZ)), the outcome for patients is still almost invariably deadly.

**Fig. 1:**
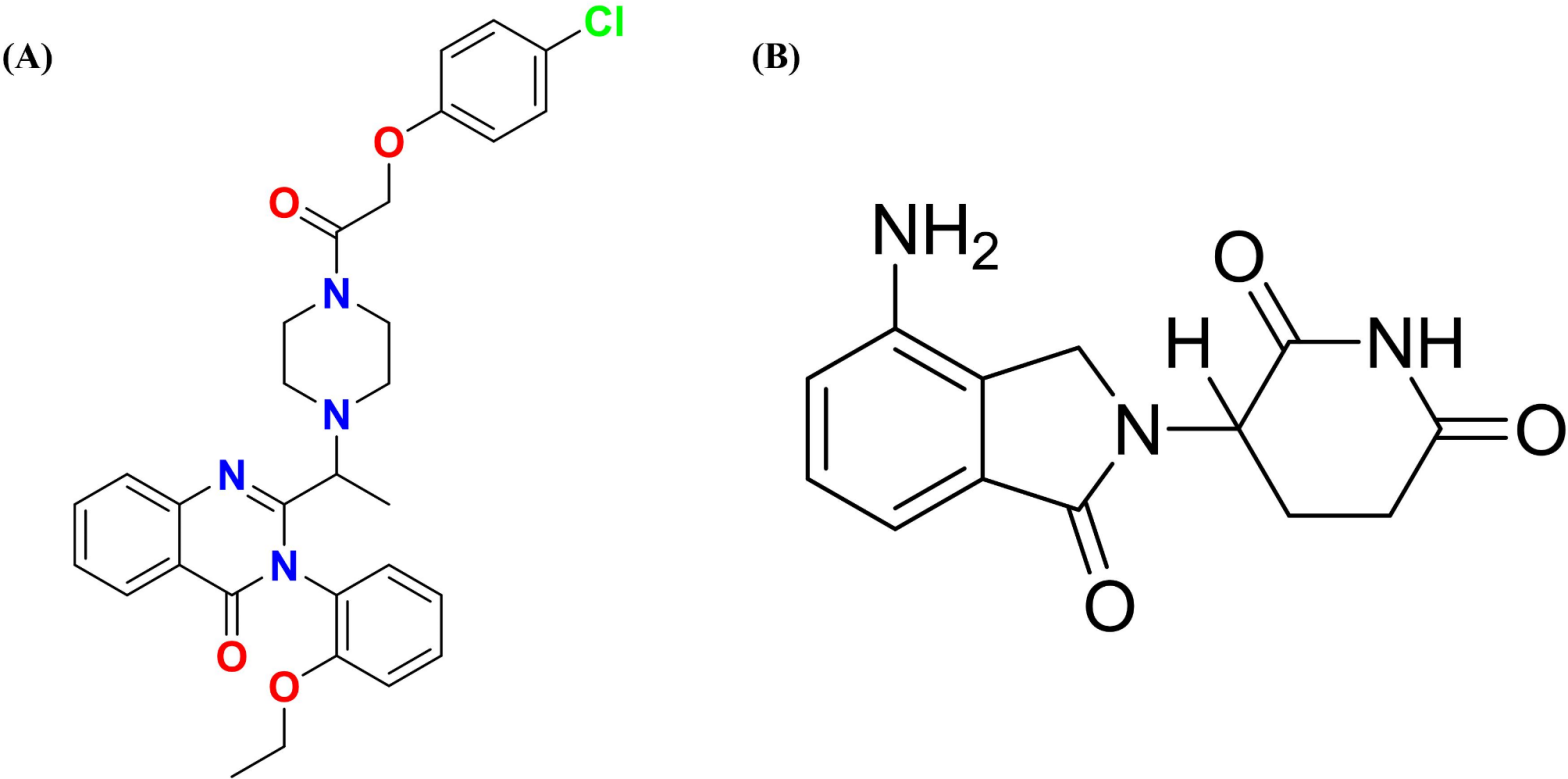
Pictorial representation of erastin and lenalidomide.

Erastin (ERT) has been mentioned more recently as a potential therapy for brain cancer through ferroptosis^2,3^. A small molecule drug known as ERT was shown to be efficient in killing human cancer cells while sparing their isogenic counterparts, normal cells. Dixon and colleagues’ further study demonstrated that ERT causes ferroptosis, a distinct category of iron-dependent controlled necrotic cell death^4^. ERT, whose chemical formula is C30H31ClN4O4, is a cell-permeable ferroptosis activator and anticancer agent with a 547 g/mol molecular weight. ERT is known by its IUPAC designation, 2-[1-[4-[2-(4-chlorophenoxy)acetyl].ethyl]piperazin-1-yl]3-(2-ethoxyphenyl)quinazolin-4-one, whose molecular makeup is depicted in Fig. 1. It falls within the BCS II class of poor solubility and high permeability with a log P value 4.8. According to reports, the ERT’s physical characteristics range from white to off-white. Through the stimulation of the voltage-dependent anion channel (VDAC), the p53 receptors, and the cystine-glutamate transport receptor (system XC−), ERT achieves its effect through ferroptosis^5,6^.

Lenalidomide (LND) is a medication that is classified as immunomodulatory. It acts as a therapeutic agent for a number of illnesses, such as blood cancer and myelodysplastic syndromes (MDS), a collection of diseases marked by aberrant synthesis of blood cells. LND works by reducing the rate at which cancer spreads and promoting the generation of healthy red blood cells. It does this by altering the bone marrow and immune system microenvironment^7,8^. There are several modes of action at work, including heightened immune cell activity, regulated cytokine and growth factor production, and anti-angiogenic actions, which prevent the creation of new blood vessels, supporting tumor growth. LND must be administered and monitored carefully under the guidance of a medical expert knowledgeable about its use and any negative effects. LND side effects include fatigue, rash, diarrhea, thrombocytopenia, anemia, neutropenia (a drop in white blood cell count), and an increased risk of blood clots^9^.

Nanopharmaceuticals are now widely recognized as a novel and effective therapeutic strategy^10,11^. A more desired release of drug profile is produced via nanoformulation, which enhances drug penetration and retention at the target location. Scientific literature has demonstrated that the enhanced targetability and better absorption of nano-formulated anti-cancer drugs contribute to their increased efficacy^12–15^. The silica (SiO2) structure of mesoporous silica nanoparticles (MSNs) resembles honeycomb cells structurally^16^. Numerous advantages are provided by their inherent porosity, flexibility in shape and size, biocompatibility, and larger surface area. Because it facilitates functional groups conjugated to MSNs, their porous structure yields a vast surface area that is especially remarkable. Drugs may be loaded into the core or onto the surface of MSNs via several practical techniques, including covalent binding, hydrophobic interactions, and electrostatic adsorption. ERT and LND were loaded into the MSNs using a sol-gel approach^17^. Chemical structure of ERT and LND is illustrated in Fig. 1.

This work aimed to stablish and verify a simple, quick, sensitive, accurate, and selective RP-HPLC technique for measuring ERT and LND that is co-loaded into MSNs. Furthermore, a pharmaceutical product’s quality characterization is required, with the user’s protection and efficiency being the primary concerns. Because of this, producing a pharmaceutical product of the highest calibre requires extensive study. The “one-factor-at-a-time” (OFAT) strategy, which changes one independent factors at a time until an appropriate response is discovered, is frequently necessary when developing a technique since it takes a lot of work^18^. The OFAT technique may recommend the right methodology, but it only offers a general knowledge of the potential and repeatability of the procedure. Conversely, employing the Analytical Quality-by-Design (AQbD) methodology offers a methodical screening procedure for analytical method development. Several pharmaceutical organizations have shown interest in employing AQbD to develop analytical procedures for various active moieties due to its many advantages. The desired standard of pharmaceutical quality has been reached since the founding of AQbD and the application of its ideas for establishing analytical techniques. The AQbD methodology is more suitable to use throughout method development than during method validation, which confirms that the analytical method is appropriate for future use. By facilitating the scientific and risk-based identification of primary sources of variation, the use of AQbD principles enhances and strengthens analytical procedures. Moreover, developing degraded peaks under harsh circumstances or after prolonged storage compromises a method’s capability to signal stability, endangering the selectivity found on a nondegraded specimen. Stress-induced force degradation studies were developed under certain stress settings, including hydrolytic, oxidative, photo, and thermal degradation to tackle these problems^19^. This study aimed to finalize the required processes, which involved creating an RP-HPLC method for quantitatively detecting ERT and LND. The method was then optimized through the utilization of the AQbD tool. The created HPLC technique was validated as per ICH Q2 (R1) guidelines, and the established HPLC technique was then utilized to measure ERT and LND in the generated MSNs. The novelty of this work is that the HPLC method is not reported for the estimation of ERT and LND.

## Materials and methods

### Material and reagents

The supplier of LND (off-white powder; purity > 98%) and ERT (off-white powder; purity > 98%) was Apicore Pharmaceutical Ltd., located in Vadodara, Gujarat. Merck Ltd., Mumbai, India, provided the orthophosphoric acid (88%) and NaOH pellets (purity ≥ 98%). Finar provided 35% AR hydrochloric acid and HPLC grade organic solvent (methanol and acetonitrile) (Ahmedabad, India). In a lab, Milli-Q (Type 1 water) was produced. Finar Inc. A 0.45 μm membrane filter was acquired from Riviera Glass Pvt. Ltd. in Mumbai, India. The C18 Spherisorb ODS column (5 μm, 120 Å, 250 mm × 4.6 mm) was procured from Phenomenex (Hyderabad, India). Tetraethylorthosilicate (TEOS) was purchased from Sisco Research Laboratories Pvt. Ltd. (SRL) (Mumbai, India), and cetyltrimethylammonium bromide (CTAB) was acquired from TCI Pharmaceuticals, Tokyo Chemical Industry (India) Pvt. Ltd. (Japan).

### Method

#### Instrumentation and apparatus

The analysis was conducted using LabSolution software, and PDA detectors (SPD-20 A & SPD-M10A) were coupled to the Shimadzu HPLC system LC20-AD (Kyoto, Japan). A calibrated weighing balance (Sartorius CP225D, Sartorious AG, Goettingen, India) was used to ensure precise weighing of every chemical included in the analysis. In the glass vacuum filtration assembly unit, a 0.45 μm membrane filter was employed to filter the buffer solution. For five minutes, the solvents were degassed using an Ultrsonicator (Servewell Instruments, India). A pH meter (model: LI 617, make: ELICO Ltd) Hyderabad, India.

#### Screening of mobile phase and standard solution

Considering the chemical structure of ERT and pKa value of LND (2.31). A buffer solution with ammonium acetate in the range of 3.8 to 5.8 was used for the optimization process. The carboxylic group in its structure of ERT and LND will ionize within the ammonium acetate buffer’s range. Ionization causes carboxylic acid to split into carboxylate ions. Spherisorb ODS C_18_ was selected as a stationary phase because octadecylsilane bonded to silica and provided the hydrophobic interaction. This will help to retain the drug in the column.

#### Preparation of standard solution for the drug

Subsequently, 10 mg of ERT and LND were dissolved in 100 µL of DMSO to produce a concentration of 1,000 µg/mL for the primary stock solutions. Methanol was added along with volume to reach 10 mL. The drug’s solution was then vortexed for a duration of two minutes. In a later step, the working stock solution was made by diluting the primary stock solution.

#### Selection of critical independent variables

The independent factors that have been chosen impact the drug’s peak characteristics, peak area, drug Rt, and resolution, as per the literature review. The buffer pH, injection volume, flow rate, and buffer ratio are the independent variables^20–22^. Furthermore, once a workable range for each variable was established, an OFAT analysis was performed to determine the independent factors significantly impacting the method outcomes^23,24^.

#### DoE supported Box-Behnken design using HPLC mothed development

The important parameters that result in the effective elution of ERT and LND must be determined and analyzed to develop the best chromatographic method for analyte quantification. If there was no experimental design, a lot of costly and time-consuming tests may be conducted. Accurate experimental values need fine-tuning of the identified critical variables as part of an improved methodology. To optimize values for these critical HPLC parameters, a detailed understanding of the chemistry of each component and how it interacts with the others is required. An approach to comprehending these various degrees of complexity is to use the DoE mathematical technique. DoE uses different response surface types to help find the sweet spot in parameter space where a consistent response might be generated. Box Behnken Design (BBD) and Central Composite Design (CCD) are two possible approaches^25,26^. The influence of independent and interaction variables was evaluated using a quadratic design. The chromatographic conditions were optimized using the BBD model. Buffer phase ratio (X1), buffer pH (X2), flow rate (X3) and injection volume (X4) were the independent factors. The dependent factors were Rt of ERT (Y1), area of ERT (Y2), Rt of LND (Y3), area of LND (Y4), resolution (Y5), respectively.

#### Method validation as per ICH guidelines

According to ICH Q2 (R1) requirements, the designed and optimized procedure for the ERT and LND was validated^27^.

#### System suitability

This is useful in determining out whether the system is appropriate for the method’s development. In this validation parameter, six ERT and LND samples were injected at 1 µg/mL concentrations. An evaluation was conducted on the tailing factors (Tf), Rt, and theoretical plate (Tp).

#### Specificity and selectivity

Selectivity and specificity studies establish of chromatographic conditions to assess a drug in the presence of different mediums, such as matrix, impurities, degradants, etc. The parameter affects identification (content or potency), assay, and purity testing. Stress-induced forced degradation and matrix interference tests were performed to determine the specificity of the best approach for LND and ERT.

#### Linearity

The linearity is used to check the ability of responses that are proportional to the drug concentration. The standard solution of ERT and LND have been prepared with the concentration range of (6.25–125 ng/mL) and (60-1250 ng/mL), respectively. All the samples were injected in the six replicates. The linearity curve was plotted between concentrations vs. peak area.

#### Accuracy

The accuracy of the developed RP-HPLC method was determined using the recovery method wherein known concentrations of ERT and LND were spiked at 3 different concentrations (80%, 100%, and 120%). All the samples for the study were prepared from the standard sample. All the samples are injected in the triplicates^28^. The % accuracy was calculated using the Eq. (1).

1 $$\:Percentage\:accuracy=\left(\frac{Percent\:recovery\:of\:test\:sample}{Percent\:recovery\:of\:reference\:standard}\right)\times\:100$$

#### Precision

The precision is demonstrated by determining the closeness in responses of the samples under the specified conditions. This test is used to verify whether, when method development is finished, comparable samples will yield the same results when the technique is applied. Repeatability, also known as an intra-day precision, was conducted by repeatedly analyzing a sample of ERT and LND over a day. To achieve inter-day precision, a sample of ERT and LND was frequently evaluated over the course of two continuous days. The proposed method’s precision is expressed in terms of %CV for peak area at individual QC sample.

#### LOD and LOQ

The established technique’s limit of detection (LOD) is the lowermost detectable or differentiable quantity of analytes in the sample under certain experimental conditions. The limit of quantification (LOQ) is the lowest quantity of drug in a sample that can be measured with enough precision and accuracy, as per the approved technique. The subsequent equations were used to determine the LOD (Eq. 2) and LOQ (Eq. 3) of the analytical method developed to measure ERT and LND. Here, σ is the standard deviation of the response, and S is the slope of the calibration curve.

2 $$\:LOD=\frac{3.3\:\sigma\:}{s}$$

3 $$\:LOQ=\frac{10\:\sigma\:}{s}$$

#### Robustness

The sensitivity of the current method was checked by gradually adjusting the chromatographic settings, and it has been demonstrated to be dependable in common scenarios^25^.

1. Change in the pH 5.75 and 5.85.
2. Change in the temperature 24 and 26.
3. Change in the flow rate 0.7 and 0.9.
4. Change in the injection volume 9 and 11.
5. Change in the wavelength + 1 and − 1.

#### Stability studies

Stability studies of the ERT and LND were check on the different conditions.

#### Bench-top studies

To evaluate the benchtop stability of the ERT and LND, the sample with a concentration of 1 µg/mL was stored at ambient temperature and analyzed after 24 h. The observations were compared with newly prepared samples^29^. The similarity index was calculated using Eq. 4.

4 $$\:Similarity\:index=\frac{peak\:area\:of\:old\:standard\:\times\:Amount\:of\:new\:standard}{Average\:peak\:area\:of\:new\:standard\:\times\:amount\:of\:old\:standard}$$

#### Stress-induced forced degradation studies

The stability of the established method was assessed by introducing a standard drug solution containing 1 µg/mL of ERT and LND to various kinds of stress conditions in accordance with ICH recommendations. We have selected the 1 µg/mL of drugs for the stress induced forced study as per the literature survey^25,30,31^. The oxidative hydrolysis (3% H2O2), photolytic degradation^32^, thermal degradation at 40 °C, acidic degradation (0.1 N HCl and 1 N HCl)^29^, and basic degradation (0.1 N NaOH and 1 N NaOH) were the conditions under which the degradation experiments under stress were conducted^33,34^. A 1 mL standard stock solution of ERT and LND at a concentration of 1 µg/mL was employed for every degradation experiment^35^. 1 mL of each stressor was added to the drug solution and then heated to 60 °C for around 24 h.

#### Application of developed and validated HPLC method

The final HPLC method was applied to check the ERT and LND concentration from the prepared MSNs.

#### Fabrication of MSNs

MSNs were fabricated using the sol-gel technique. During this procedure, TEOS was gradually added, along with CTAB, NaOH, and deionized water. For two hours, the mixture was agitated at 800 rpm and 78 °C. Three washing cycles, a 10-minute centrifugation at 10,000 rpm, and a 24-hour hot air oven drying procedure at 600 °C were all applied to the formulation. Afterward that, the formulation was kept in a muffle furnace for three hours at 550 °C to be calcined. Dependent factors, including polydispersity index, zeta potential, and particle size, were used to produce the placebo MSNs.

#### Preparation of erastin and lenalidomide loaded MSNs

The ERT and LND were loaded into MSNs using the incubation method. To put it briefly, 3 mg of ERT and 6 mg of LND were combined with 1 mL of ethanol. Furthermore, this mixture was incubated in an ethanol solution that included two mg of MSNs. The MSNs were then sonicated for five minutes, and their dispersion was agitated for 24 hour at an ambient temperature of 500 rpm. Then, the dispersion was centrifugated at 15,000 rpm for 10 minute. The following step involved washing the precipitate three times with ethanol. Finally, the synthetically synthesized ERT-LND-loaded MSNs (ERT-LND@MSNs) have been characterized and optimized with dependent parameters such as particle size, zeta potential, and PDI^36–38^.

#### Calculation of % entrapment efficiency (% EE) and % drug loading (% DL)

The dispersion was centrifugated for 10 minute at 15,000 rpm after loading, and the amount of ERT and LND that remained in the supernatant (unentrapped ERT and LND) was determined using the suggested RP-HPLC technique. To find the percentages of EE and DL of ERT and LND in MSNs, Eqs. 5 and 6 were utilized^39–41^.

5 $$\:\text{\%}\:\text{E}\text{n}\text{t}\text{r}\text{a}\text{p}\text{m}\text{e}\text{n}\text{t}\:\text{E}\text{f}\text{f}\text{i}\text{c}\text{i}\text{e}\text{n}\text{c}\text{y}\:\left(\text{\%}\text{E}\text{E}\right)=\frac{intial\:amount\:of\:drug-amount\:of\:drug\:in\:supernatant}{initial\:amount\:of\:drug}\times\:100\%$$

6 $$\:\%\:Drug\:Loading\:\left(\text{\%}\text{D}\text{L}\right)=\frac{intial\:amount\:of\:drug-Amount\:of\:drug\:in\:supernatant}{Total\:weight\:of\:formulation}\times\:100\%$$

#### In-vitro drug release study

The release behaviour of the developed ERT-LND@MSNs was placed in dialysis membrane (Molecular weight 12000 Da). The release of ERT and LND from the fabricated MSNs at pH 5.5. and 7.4 was evaluated at a temperature of 37 °C. The release study was performed with 1.5 mg of LND and 0.75 mg of ERT loaded MSNs (ERT-LND@MSNs). The ERT-LND@MSNs was suspended in 2 mL of PBS (0.1 M, pH 7.4 and pH 5.5) were first placed into a dialysis bag and dialyzed against 50 mL of different buffer medium as mentioned. At predetermined time intervals, 1 mL of aliquot was withdrawn, and an equal volume of fresh buffer was added. The concentration of ERT and LND was analysed using the validated optimized RP-HPLC method^42^.

#### Scanning electron microscopy (SEM)

SEM was used to evaluate the synthesized MSNs’ surface characteristics. The MSN was ready for an SEM investigation on an aluminum counterfoil. A gold sputtering coater was used to apply a thin layer of gold, and an EVO MA18 Zeiss scanning electron microscope was used to examine the sample^43–45^.

#### Greenness evaluation of the developed HPLC method

The greenness of the analytical technique was evaluated utilizing the AGREE software (Analytical GREEnness Metric Approach and Software)^46^ and GAPI (Green Analytical Procedure Index) software^47^. The AGREE technique considers twelve factors, each given a score between 0 and 1, with higher numbers denoting a greener method. The GAPI involves 15 factors, each representing various features of the analytical procedure, such as sampling, reagents and solvents used, sample preparation, instruments, and waste generated. These techniques is designed to check the effect of analytical method on the environment.

## Results and discussion

### Method development

The UV-Vis spectrophotometer was used to determine the ERT and LND absorbance maximum, which was found to be 219 nm. This wavelength was chosen because it provides optimal sensitivity and accuracy for both compounds simultaneously.

### Selection of mobile phase and stationary phase

As we developed the analytical method for ERT and LND, we explored into a number of additional possible mobile phases. ERT was shown to elute in the dead volume with a considerable Tf during the early trials when acetonitrile/methanol was combined in different ratios with a phosphate buffer. LND was not showing peak. Next, we tried an alternative approach using an ammonium acetate buffer and acetonitrile/methanol as the mobile phase. Unfortunately, for both ERT and LND, this combination resulted in poor symmetry and a plate count of fewer than 2000. The previous experiment changed the mobile phase’s methanol and ammonium acetate ratio. This ratio elutes both drugs after 3 min. The peak properties such Tf of less than 2 and a Tp count of more than 2000 were observed. Initially, we employed a standard C_18_ stationary phase (250 mm × 4.6 mm, 5 μm particle size, Waters), which produced ERT and LND parameter peaks that fulfilled the required standards. To further enhance and optimize the chromatographic conditions, the DoE technique was applied.

### DoE supported HPLC method optimization using BBD

The optimization approach employed in this method development directly impacted the independent parameters that influenced the chromatographical outcomes. A lack of complete understanding of these important approach factors weakens the method’s robustness and raises the chances that it will not function as intended in many scenarios. It is important to identify and check these independent factors to develop an accurate and precise method for determining ERT and LND.

In this work, the independent variables were adjusted by three-level BBD. The independent factors were buffer ratio (X1), buffer pH(X2), flow rate (X3), and injection volume (X4). The higher and lowest limits of the model were screened using the OFAT test. The upper limit for the buffer ratio was 80%, and the lower limit was 60%. The pH of the buffer varied between 3.8 (the lower limit) and 5.8 (the higher limit), while the flow rate was between 0.8 mL/min (the lower limit) and 1 mL/min (the upper limit). 5 µl (lowest limit) to 10 µl (highest limit) of injection volume were covered. However, relying only on OFAT-based approaches might be challenging and time-consuming. Thus, applying BBD and alternative response surface designs accelerates the optimization procedure and enhances the development of a dependable analytical technique.

A total of 46 trial runs were conducted using a single center point. The details of these trial run and the corresponding responses are shown in Table 1. The results of the ANOVA analysis indicate significant interactions between the independent variables, which are displayed in Table 2.

**Table 1:** The experimental design suggested by the DoE and their responses.

Run	X1	X2	X3	X4	Y1	Y2	Y3	Y4	Y5
1	70	5.8	0.8	7.5	4.666	970,922	6.2265	52,047	3.0385
2	70	4.8	0.9	7.5	4.184	862,051	5.623	45939.5	3.2615
3	60	4.8	0.9	7.5	3.772	977,741	5.1525	5257.5	3.6315
4	80	5.8	0.9	7.5	4.898	859,055	9.7105	45,300	7.068
5	70	3.8	0.8	7.5	4.6865	503,180	6.258	32820.5	3.467
6	60	3.8	0.9	7.5	3.77	582,026	6.499	32,045	5.025
7	80	4.8	0.9	10	4.9125	1,119,482	9.7315	57187.5	7.764
8	60	4.8	0.8	7.5	4.2205	1,111,386	5.722	5855	3.636
9	70	3.8	1	7.5	3.738	422,454	4.9225	25,388	3.029
10	70	4.8	0.9	7.5	4.158	877,836	5.541	46600.5	3.212
11	60	4.8	1	7.5	3.3945	885,153	4.5855	5837.5	2.8925
12	80	4.8	0.9	5	4.9155	566,709	9.8205	28,439	7.305
13	80	4.8	0.8	7.5	5.532	1,009,797	11.0815	55137.5	7.9145
14	70	4.8	0.9	10	4.1695	1,093,483	5.565	60,079	3.188
15	70	5.8	1	7.5	3.7445	786,575	4.996	42843.5	2.973
16	80	4.8	0.9	7.5	4.8605	894,914	9.387	48843.5	7.4005
17	70	4.8	0.8	5	4.6615	611,853	6.174	33136.5	3.271
18	70	4.8	0.8	7.5	4.6785	979,255	6.2435	53,435	3.2645
19	60	4.8	0.9	10	3.775	1,221,693	5.1255	8147	3.447
20	70	5.8	0.9	10	4.1545	1,077,532	5.5175	58,999	2.9735
21	70	4.8	0.9	7.5	4.17	875,914	5.59	46547.5	3.2805
22	70	4.8	0.9	7.5	4.173	876,648	5.5865	48,518	3.2315
23	80	4.8	0.9	7.5	4.94	903,360	9.9505	49399.5	8.116
24	80	4.8	1	7.5	4.449	669,244	8.9295	35509.5	8.343
25	70	4.8	0.8	10	4.6945	1,224,380	6.2945	69170.5	3.2805
26	70	3.8	0.9	5	4.157	295,555	5.497	18,893	3.388
27	70	4.8	0.9	7.5	4.1775	876,715	5.609	47411.5	3.3115
28	70	4.8	1	5	3.7645	497,536	5.0555	28401.5	3.27
29	70	5.8	0.9	7.5	4.154	879,031	5.5275	50,111	3.018
30	70	4.8	1	7.5	3.7665	794,985	5.0595	44065.5	3.2615
31	70	4.8	0.9	5	4.132	547,058	5.4105	29219.5	3.1185
32	70	3.8	0.9	10	4.159	574,275	5.504	36,853	3.234
33	70	4.8	0.9	5	4.1505	546,624	5.4915	27,992	3.288
34	70	5.8	0.9	5	4.1575	547,269	5.536	29611.5	3.0455
35	80	3.8	0.9	7.5	4.9085	395,322	9.5565	28,281	7.813
36	70	4.8	0.9	10	4.1675	1,090,577	5.579	60,209	3.2845
37	70	4.8	1	10	3.755	990,123	5.01	53984.5	3.1405
38	70	5.8	0.9	7.5	4.121	872,208	5.3785	49621.5	2.8435
39	70	4.8	0.8	7.5	4.645	981,009	6.0845	54,828	3.118
40	70	3.8	0.9	7.5	4.1595	472,166	5.498	43,940	3.251
41	70	4.8	1	7.5	3.7265	793,229	4.878	43642.5	3
42	60	4.8	0.9	7.5	3.7435	986,058	5.0205	7173	3.295
43	70	4.8	0.9	7.5	4.1555	885,366	5.525	47817.5	3.22
44	60	4.8	0.9	5	3.7645	616,162	5.1565	3331.5	4.043
45	70	3.8	0.9	7.5	4.109	448,092	5.3015	28632.5	3.237
46	60	5.8	0.9	7.5	3.75	967,557	5.0565	2368.5	3.925

**Table 2:** ANOVA results of Box-Behnken design.

Response	Rt of ERT (Y1)		Peak area of ERT (Y2)		Rt of LND (Y3)		Peak area of LND (Y4)		Resolution (Y5)	
F-value	85.95		12.19		7.26		6.67		3.22	
p-value	Model	< 0.0001	Model	< 0.0001	Model	< 0.0001	Model	< 0.0001	Model	0.0049
	A	< 0.0001	A	0.0769	A	< 0.0001	A	< 0.0001	A	< 0.0001
	B	0.9180	B	< 0.0001	B	0.8068	B	0.0811	B	0.5350
	C	< 0.0001	C	0.0044	C	0.0212	C	0.1099	C	0.8502
	D	0.8359	D	< 0.0001	D	0.9667	D	< 0.0001	D	0.9419
	AB	0.9628	AB	0.7610	AB	0.4742	AB	0.0538	AB	0.9012
	AC	0.2125	AC	0.6568	AC	0.6482	AC	0.4077	AC	0.6823
	AD	0.9472	AD	0.8374	AD	0.9792	AD	0.3134	AD	0.7125
	BC	0.8946	BC	0.6871	BC	0.9623	BC	0.9401	BC	0.8964
	BD	0.9804	BD	0.3309	BD	0.9908	BD	0.6283	BD	0.9771
	CD	0.8349	CD	0.6411	CD	0.9405	CD	0.6579	CD	0.9612
R^2^	0.9609		0.7769		0.6747		0.6559		0.4790	
Adjusted R^2^	0.9497		0.7131		0.5818		0.5575		0.3302	

### Impact of independent factors on retention time (Rt) of erastin (Y1)

**Fig. 2:**
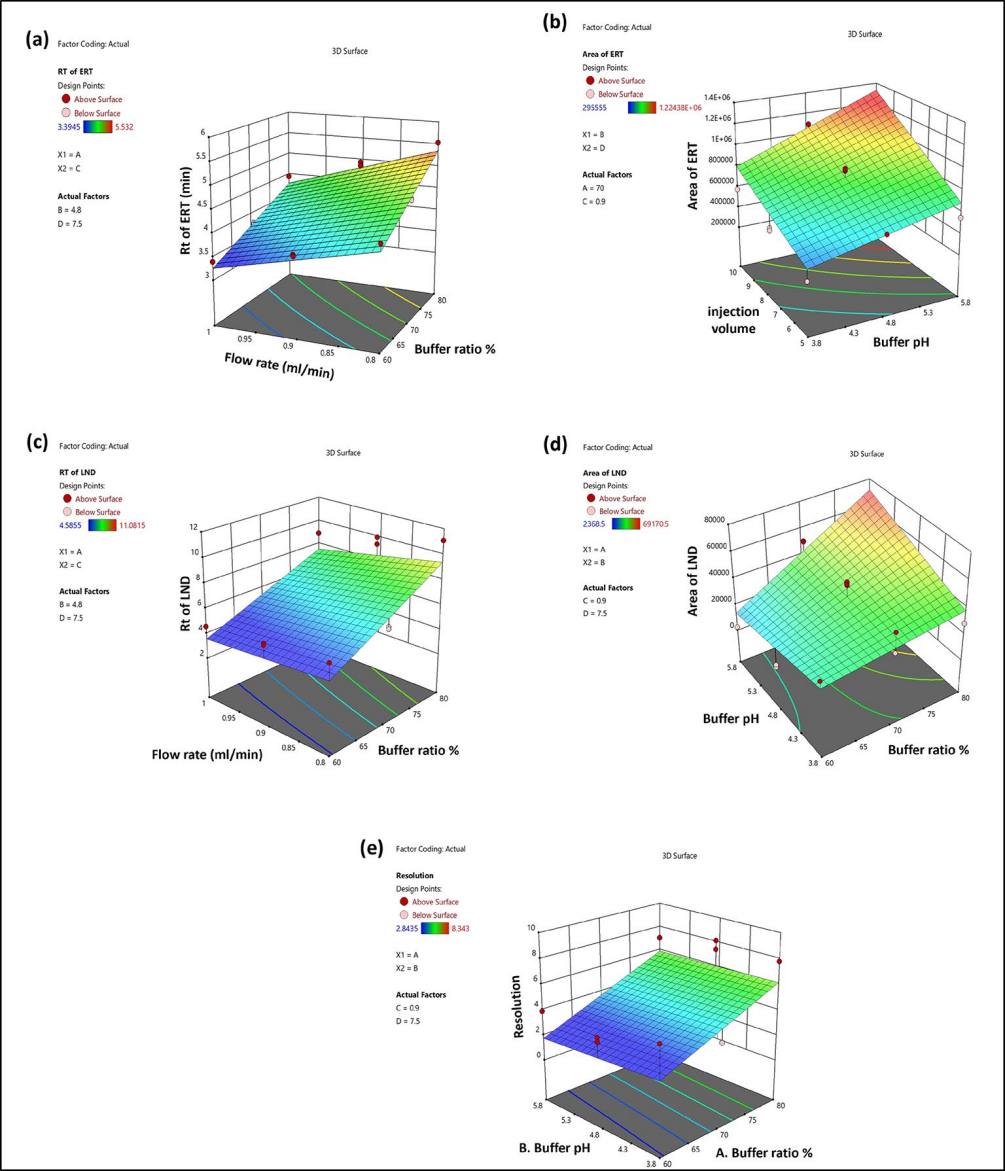
The 3D surface response graphic illustrating how independent factors have an impact: (**a**) on the Rt of Ert, (**b**) on the peak area of ERT, (**c**) on the Rt of LND, (**d**) on the peak area of LND, (**e**) resolution.

**Fig. 3:**
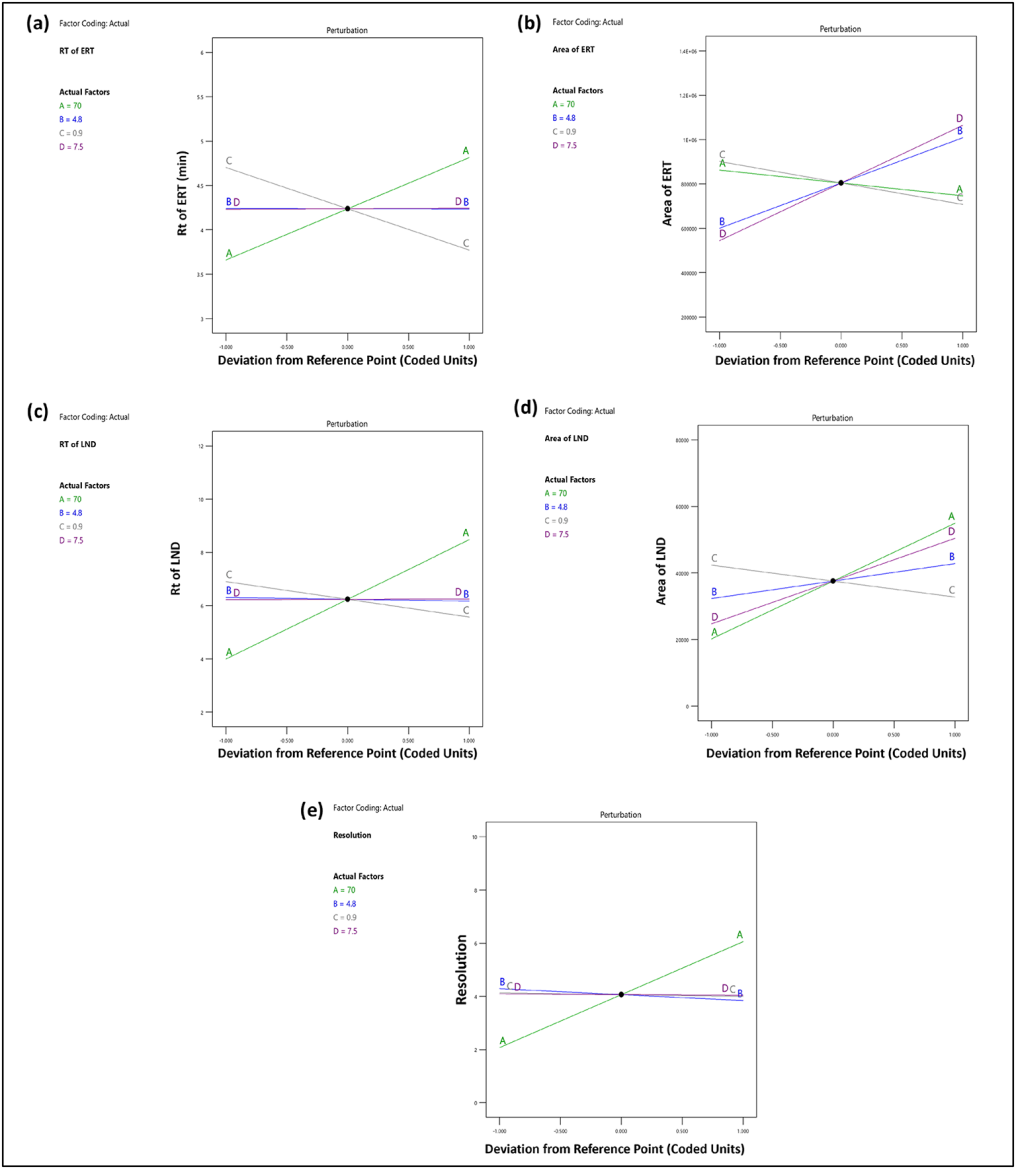
A perturbation diagram that illustrates how independent variables interact (**a**) on the Rt of ERT, (**b**) on the peak area of ERT, (**c**) on the Rt of LND, (**d**) on the peak area of LND, (**e**) resolution.

The quadratic equation (Eq. 7) and ANOVA analysis were used to demonstrate the effect of the independent factors X1 and X3 on the Rt of ERT. On the other hand, the Rt was less affected by the X4 and X2. More precisely, a lower flow rate and a larger buffer ratio resulted in an extended Rt for ERT. In this study, we have increased the X1 and X3 levels from 60 to 80% and from 0.8 to 1 mL/min, which resulted in a shift in the Rt from 3.72 to 4.94 min for ERT. The effects of these independent factors, X1 and X3, are depicted in the 3D and perturbation graphs in Figs. 2a and 3a.

7 $$\begin{aligned} Y1{\text{ }} & = {\text{ }} + 4.23787{\text{ }} + {\text{ }}0.576625{\text{ }} \times {\text{ }}A{\text{ }}{-}{\text{ }}0.002625{\text{ }} \times {\text{ }}B{\text{ }} + {\text{ }} - 0.465375{\text{ }} \times {\text{ }}C{\text{ }} \\ & + {\text{ }}0.00528125{\text{ }} \times {\text{ }}D{\text{ }} + {\text{ }}0.002375{\text{ }} \times {\text{ }}AB{\text{ }}{-}{\text{ }}0.06425{\text{ }} \times {\text{ }}AC{\text{ }}{-}{\text{ }}0.003375 \\ & \times {\text{ }}AD{\text{ }} + {\text{ }}0.00675{\text{ }} \times {\text{ }}BC{\text{ }}{-}{\text{ }}0.00125{\text{ }} \times {\text{ }}BD{\text{ }}{-}{\text{ }}0.010625{\text{ }} \times {\text{ }}CD \\ \end{aligned}$$

### Impact of independent factors on the peak area of erastin (Y2)

Quadratic equation (Eq. 8) generated by ANOVA analysis revealed that X2, X3 and X4 significantly affected the Y2. However, the Y2 mainly was unaffected by the buffer ratio (X1). Factor X2 and X4 will increase and X3 will decrease so ERT’s peak area will increase. By changing the factors X2, X3, and X4, the peak area was between 295,555 and 1224379.5 for ERT. The effects of these independent variables, X2, X3 and X4, are depicted in the 3D and perturbation graphs in Figs. 2b and 3b.

8 $$\begin{aligned} Y2{\text{ }} & = {\text{ }} + {\text{ }}804773{\text{ }}{-}{\text{ }}58118.4{\text{ }} \times {\text{ }}A{\text{ }} + {\text{ }}204192{\text{ }} \times {\text{ }}B{\text{ }}{-}{\text{ }}97030.1{\text{ }} \times {\text{ }}C{\text{ }} + {\text{ }}260174{\text{ }} \times {\text{ }}D{\text{ }} + {\text{ }}19550.5{\text{ }} \times {\text{ }}AB{\text{ }}{-}{\text{ }}28580{\text{ }} \\ & \times {\text{ }}AC{\text{ }} + {\text{ }} - 13189.5{\text{ }} \times {\text{ }}AD{\text{ }}{-}{\text{ }}25905.3{\text{ }} \times {\text{ }}BC{\text{ }} + {\text{ }}62885.6{\text{ }} \times {\text{ }}BD{\text{ }}{-}{\text{ }}29984.9{\text{ }} \times {\text{ }}CD \\ \end{aligned}$$

### Impact of independent factors on retention time (Rt) of lenalidomide (Y3)

The quadratic equation (Eq. 9) created by ANOVA analysis revealed that independent factors X1 and X3 significantly affected the Rt of LND. The X2 and X4 have shown less effect on the Rt of LND. The increase in X1 and decrease in X3 increases the Rt of LND. In this study, we have increased the X1 and X3 levels from 60 to 80% and from 0.8 to 1 mL/min, which resulted in a shift in the Rt from 4.58 to 11.08 min for ERT. The effects of these independent factors, X1 and X3, are depicted in the 3D and perturbation graphs in Figs. 2c and 3c.

9 $$\begin{aligned} Y3{\text{ }} & = {\text{ }} + {\text{ }}6.23845{\text{ }} + {\text{ }}2.24059{\text{ }} \times {\text{ }}A{\text{ }}{-}{\text{ }}0.0679687{\text{ }} \times {\text{ }}B{\text{ }}{-}{\text{ }}0.6655{\text{ }} \times {\text{ }}C{\text{ }} + {\text{ }}0.0115938{\text{ }} \times {\text{ }}D{\text{ }} + {\text{ }}0.399125{\text{ }} \times {\text{ }}AB \\ {\text{ }} & {-}{\text{ }}0.253875{\text{ }} \times {\text{ }}AC{\text{ }}{-}{\text{ }}0.0145{\text{ }} \times {\text{ }}AD{\text{ }} + {\text{ }}0.02625{\text{ }} \times {\text{ }}BC{\text{ }}{-}{\text{ }}0.006375{\text{ }} \times {\text{ }}BD{\text{ }}{-}{\text{ }}0.0415{\text{ }} \times {\text{ }}CD \\ \end{aligned}$$

### Impact of independent factors on the peak area of lenalidomide (Y4)

The ANOVA analysis generate the quadratic equation (Eq. 10) showed that independent parameters that significantly impacted the LND peak area were the X1, X2, X3, and X4. Factor X1, X2 and X4 will increase and X3 decreases so the peak area of LND will increase. By changing the factors X1, X2, X3, and X4, the peak area was between 2368.5 and 69170.5 for LND. The effects of these independent variables, X1, X2, X3 and X4, are depicted in the 3D and perturbation graphs in Figs. 2d and 3d.

10 $$\begin{aligned} Y4{\text{ }} & = {\text{ }} + {\text{ }}37584.2{\text{ }} + {\text{ }}17380.2{\text{ }} \times {\text{ }}A{\text{ }} + {\text{ }}5253.06{\text{ }} \times {\text{ }}B{\text{ }}{-}{\text{ }}4797.34{\text{ }} \times {\text{ }}C{\text{ }} + {\text{ }}12850.3{\text{ }} \times {\text{ }}D{\text{ }} + {\text{ }}11673.9{\text{ }} \times {\text{ }}AB{\text{ }} \\ & {-}{\text{ }}4902.63{\text{ }} \times {\text{ }}AC{\text{ }} + {\text{ }}5983.25{\text{ }} \times {\text{ }}AD{\text{ }}{-}{\text{ }}442.75{\text{ }} \times {\text{ }}BC{\text{ }} + {\text{ }}2856.88{\text{ }} \times {\text{ }}BD{\text{ }}{-}{\text{ }}2612.75{\text{ }} \times {\text{ }}CD \\ \end{aligned}$$

### Impact of independent factors on resolution (Y5)

The response Y5 have shown the most impact from the independent factors among all the other responses that were investigated in this study. Rt difference between two neighboring peaks differ is the resolution. The quadratic equation (Eq. 11) created by ANOVA analysis showed the effect of independent factors on the resolution. The increase in X1 and X2 increases the resolution. The 3D and perturbation plots are shown in Figs. 2e and 3e.

11 $$\begin{aligned} Y5{\text{ }} & = {\text{ }} + {\text{ }}4.0678{\text{ }} + {\text{ }}1.98931{\text{ }} \times {\text{ }}A{\text{ }}{-}{\text{ }}0.222437{\text{ }} \times {\text{ }}B{\text{ }}{-}{\text{ }}0.0675313{\text{ }} \times {\text{ }}C{\text{ }}{-}{\text{ }}0.0260625{\text{ }} \times {\text{ }}D{\text{ }} + {\text{ }}0.08875{\text{ }} \\ & \times {\text{ }}AB{\text{ }} + {\text{ }}0.293{\text{ }} \times {\text{ }}AC{\text{ }} + {\text{ }}0.26375{\text{ }} \times {\text{ }}AD{\text{ }} + {\text{ }}0.093125{\text{ }} \times {\text{ }}BC{\text{ }} + {\text{ }}0.0205{\text{ }} \times {\text{ }}BD{\text{ }}{-}{\text{ }}0.03475{\text{ }} \times {\text{ }}CD \\ \end{aligned}$$

### Desirability

**Fig. 4:**
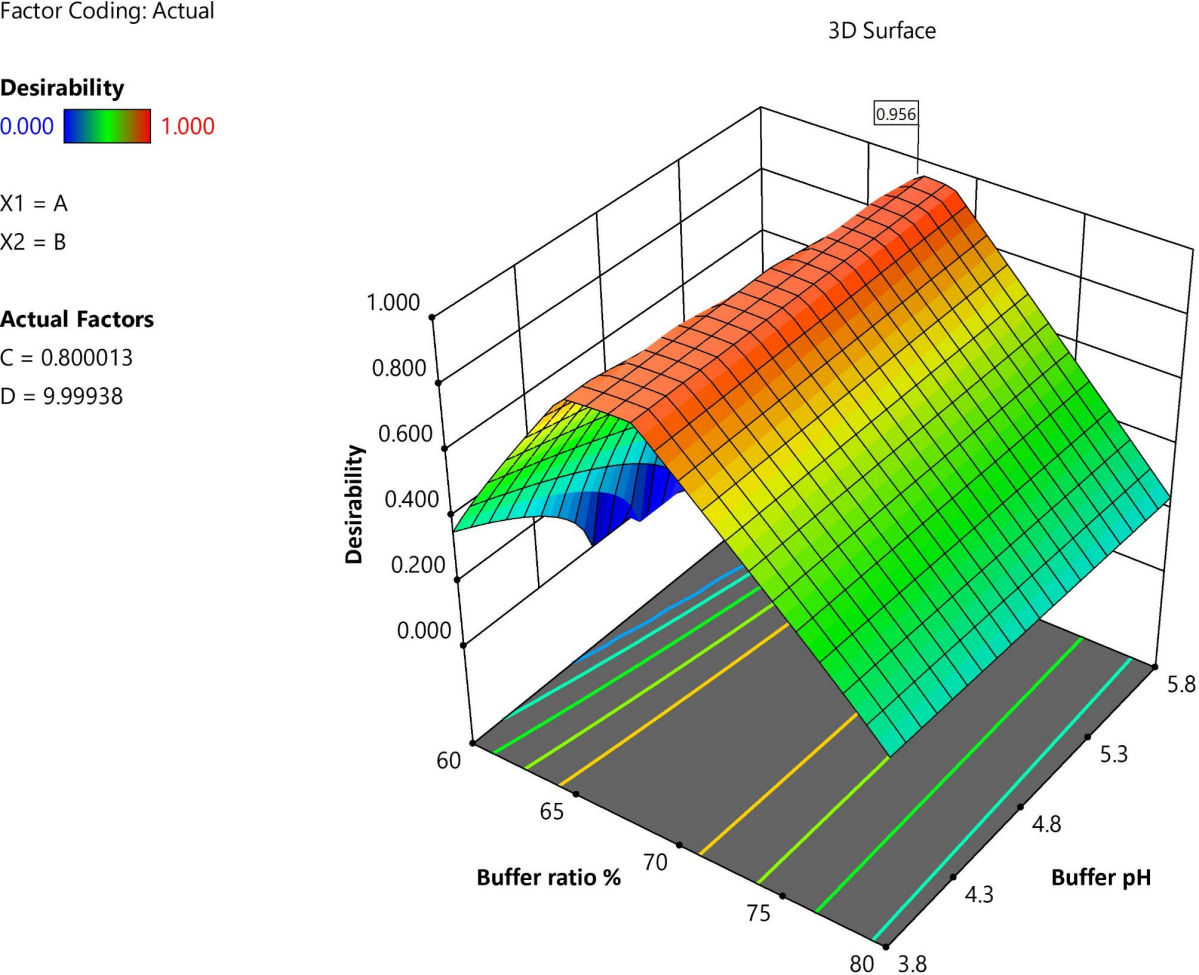
Desirability plot from the ANOVA analysis.

The previous analysis produced an optimized RP-HPLC method with a desirability value of 0.956. The final method suggested an injection volume of 10 µl, a buffer pH of 5.8, flow rate 0.8 mL/min, a buffer ratio of 68% and methanol 32%. Six repetitions of the suggested method were run in order to validate it, and the results for every response were compared to the values provided by the program. This comparison showed that the replies percentage relative inaccuracy stayed below 10%. The recommended method was then subjected to further validation in compliance with ICH Q2 (R1) requirements. The chromatogram that was obtained under ideal conditions is shown in Fig. 4.

### Method validation

The validation parameters including Rt, Tf, and Tp were calculated to check the system suitability (Table 3).

**Table 3:** Validation data of the optimized analytical method for the ERT and LND.

Parameters	Erastin	Lenalidomide
Retention time (min)	4.706	6.306
Tailing factor	1.494	1.141333
Theoretical plate	2794.112667	3074.866
Specificity	No interfering peaks at the tR of erastin and LND	
Resolution	3.801	
Linearity range (ng/mL)	6.25–125	60-1250
Regression equation	Y = 22697x + 52,629	Y = 36.678x + 1202.2
Correlation coefficient (r^2^)	0.993	0.991
LOD (ng/mL)	0.25	31.25
LOQ (ng/mL)	0.75	50
Accuracy (% Recovery)		
80%	101.177	100.447
100%	101.649	101.741
120%	99.475	99.198
Precision (% CV)		
Repeatability	0.122	0.28
Inter-day	1.882	0.61

### System suitability

The system suitability of the developed RP-HPLC method for ERT and LND estimation was validated. We calculated the Tf, resolution, Tp, and Rt. All observations were met into the accepted criteria. The system suitability observation and peak purity of the ERT and LND have showed in Table S1 Fig. S1.

### Linearity

**Fig. 5:**
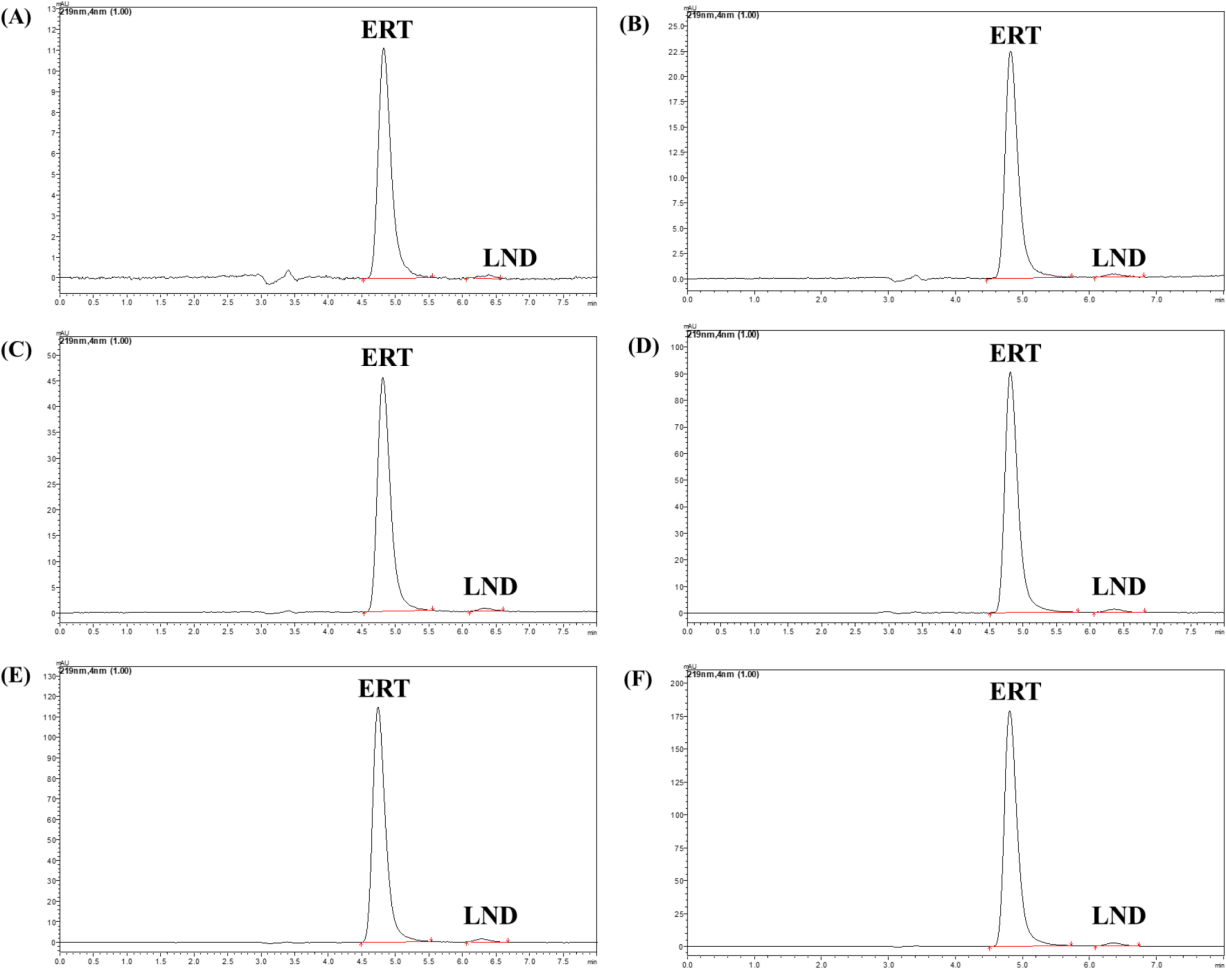
Chromatogram of the linearity sample with concentration in ng/mL (**A**) ERT 6.25 and LND 60, (**B**) ERT 12.5 and LND 120, (**C**) ERT 25 and LND 250, (**D**) ERT 50 and LND 500, (**E**) ERT 100 and LND 1000, (**F**) ERT 125 and LND 1250.

The linearity was performed for the ERT in the range of 6.25–125 ng/mL and for LND 60-1250 ng/mL. The R^2^ value for ERT and LND was calculated from the calibration curve 0.993 and 0.991, respectively. The linearity equation for the ERT was y = 22697x + 52,629, and for the LND y = 36.678x + 1202.2. The pictorial representation of the chromatogram is shown in Fig. 5 and the calibration curve in Fig. S2.

### Sensitivity

The LOD and LOQ were determined using Eqs. 1 and 2. It was determined that the ERT’ LOD and LOQ were, respectively, 0.75 ng/mL and 1.62 ng/mL. The results of LOD and LOQ for LND were, respectively, 31.25 ng/mL and 50 ng/mL.

### Accuracy

**Fig. 6:**
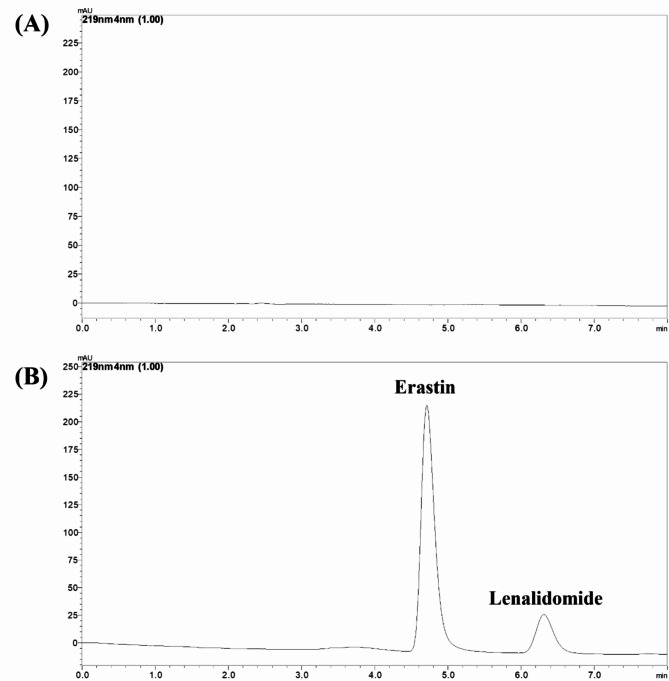
Chromatogram found at optimized conditions (**A**) Chromatogram of blank MSNs, (**B**) Chromatogram of ERT and LND loaded MSNs.

The accuracy of the developed RP-HPLC method were carried at three separate concentrations: 80%, 100%, and 120%. The percentage for ERT and LND was between 99 and 102% recovery, which is an acceptable range according to ICH guidelines. The method that was developed was exact and reliable. The optimized chromatogram is showen in Fig. 6.

### Precision

The intraday and inter-day precision of the established RP-HPLC analytical method for the ERT and LND was evaluated. The % CV for the intraday and inter-day was observed to be less than 2%.

### Robustness

We experimented with minor adjustments to the buffer pH, wavelength, injection volume, flow rate, and temperature. These chromatographic condition alterations did not result in any discernible changes to the properties of the peaks of the drugs. This implies that the existing approach for ERT and LND estimation is valid. The robustness data is shown in Table S2 and Fig. S3. 

### Bench-top stability

it was found that the recovery from the working solution was between 100 and 105%, and the percentage CV was less than 2%.

### Stress-induced forced degradation studies

**Fig. 7:**
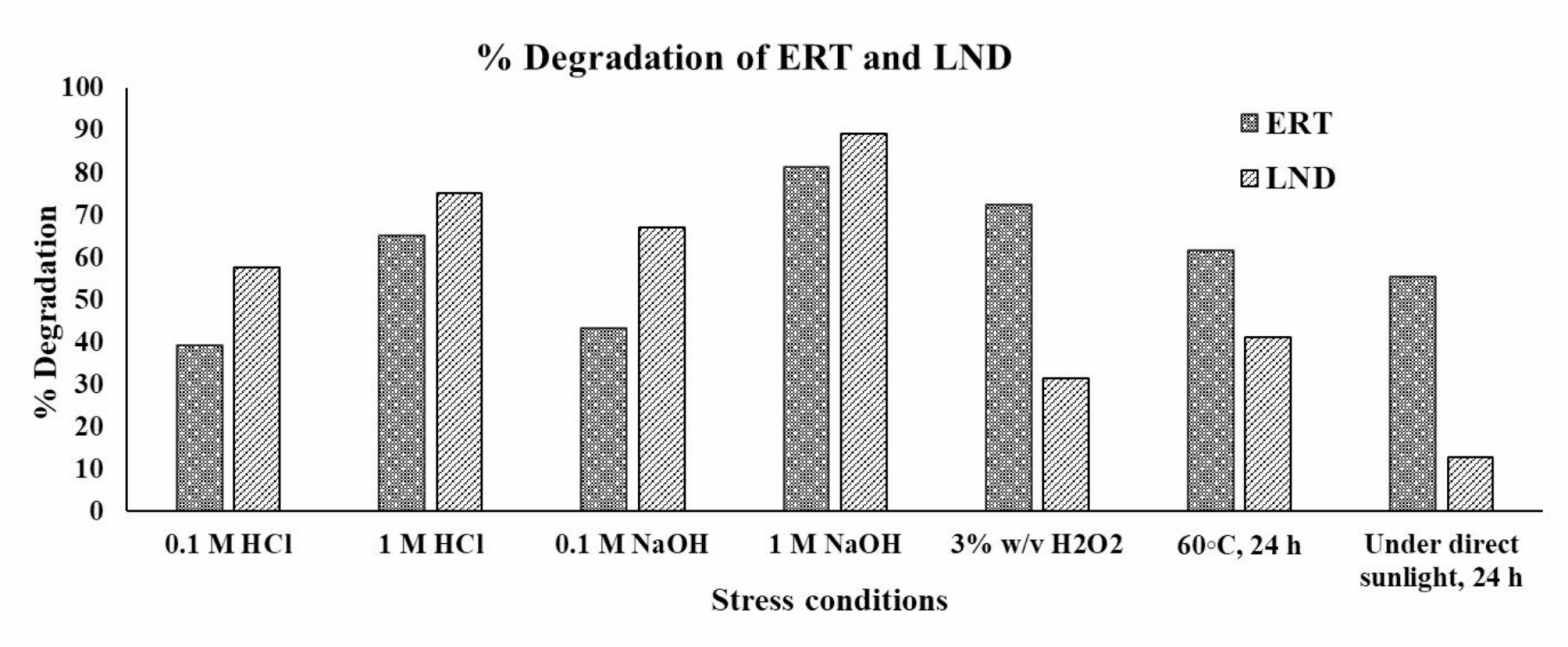
Column chart represented the stress-induced forced degradation study of ERT and LND.

The stability of ERT and LND were performed in various stress conditions to evaluate the drugs withstand the stress conditions of the formulation development. A thorough deterioration investigation was thus performed. Fig. 7 shows the impact of different stress situations and the corresponding deterioration. Degradation was more than 39% for ERT and more than 57% for LND in the 0.1 M HCl, more than 65% for ERT, and more than 75% for LND in the 1 M HCl. Degradation was more than 43% for ERT and more than 67% for LND in the 0.1 M NaOH, more than 81% for ERT and more than 89% for LND in the 1 M NaOH. Degradation was more than 72% for ERT and more than 31% for LND under oxidative conditions with H2O2. In the forced temperature (60℃) condition, degradation was more than 61% for ERT and more than 41% for the LND. In the sunlight (UV) condition, degradation was more than 55% for ERT and more than 12% for the LND. The chromatogram of the stress-induced forced degradation study is shown in Fig. S4.

### Application of the developed RP-HPLC analytical method

#### Evaluation of fabricated placebo MSNs and ERT-LND@MSNs

**Fig. 8:**
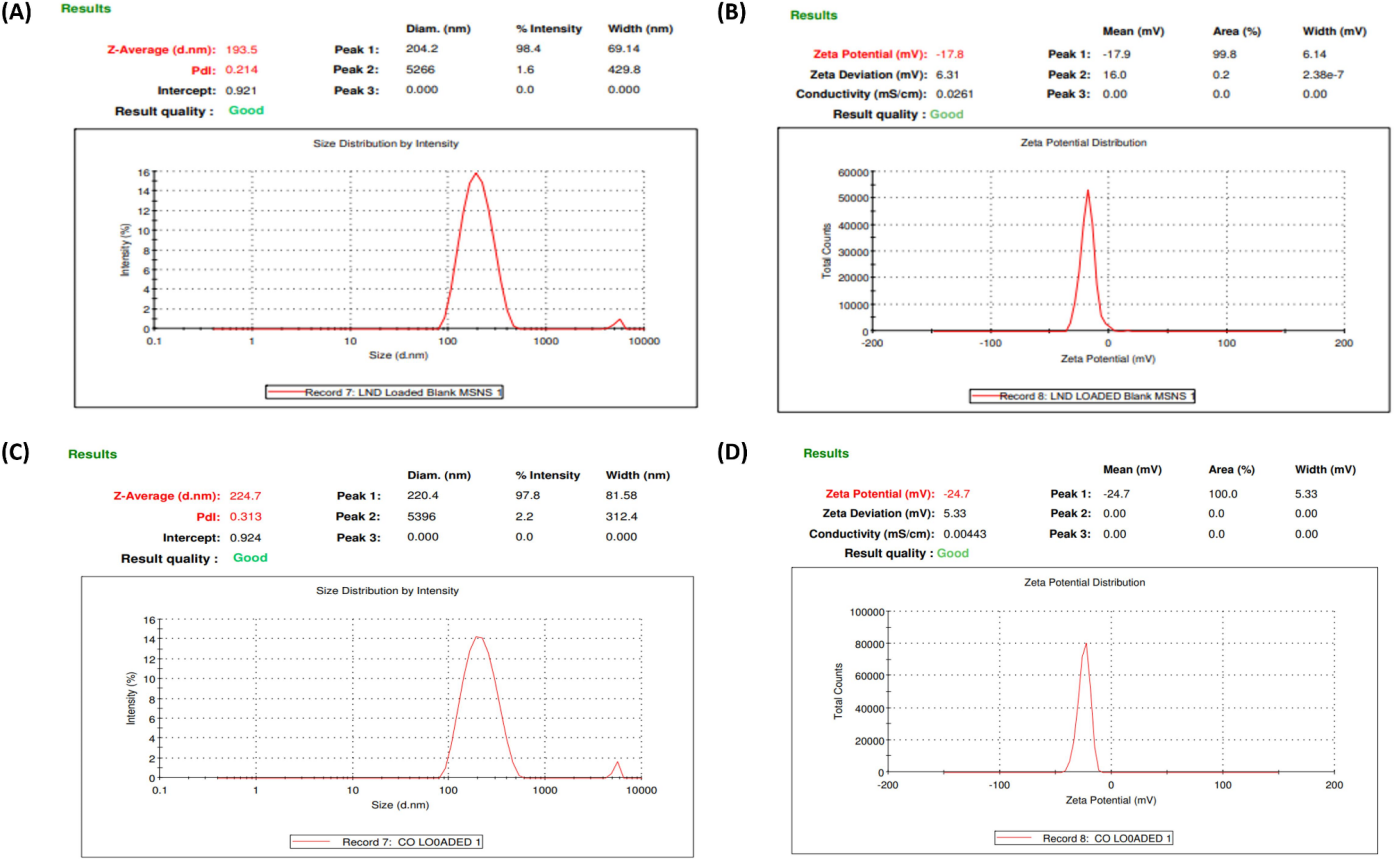
Characterization of fabricated formulation: (**A**) Particle size of placebo MSNs, (**B**) Zeta potential of placebo MSNs, (**C**) Particle size of ERT-LND@MSNs, (**D**) Zeta potential of ERT-LND@MSNs.

It was found that the average particle sizes of placebo MSNs and ERT-LND@MSNs were 193.5 nm and 224.7 nm, respectively. Notably, as seen in the figures, the placebo MSNs and ERT@MSNs had polydispersity indices of 0.214 and 0.313, demonstrating a consistent particle size distribution throughout the formulation. Zeta potentials of placebo MSNs and ERT@MSNs were determined to be −17.8 mV and − 24.7 mV, respectively Fig. 8.

#### The specificity of the validated method

It has been confirmed that the excipients do not impact the Rt of LND and ERT. The ERT and LND from the ERT-LND loaded MSNs can be quantified using the established analytical technique. The chromatograms of MSNs loaded with ERT-LND and placebo MSNs are shown in Fig. 6.

### % entrapment efficiency and % drug loading

The % EE in MSNs of ERT was found to be 72.65% and for LND was found to be 79.50. % DL in MSNs of ERT was found to be 14% and for LND was found to be respectively.

### In-vitro drug release from the ERT-LND@MSNs

**Fig. 9:**
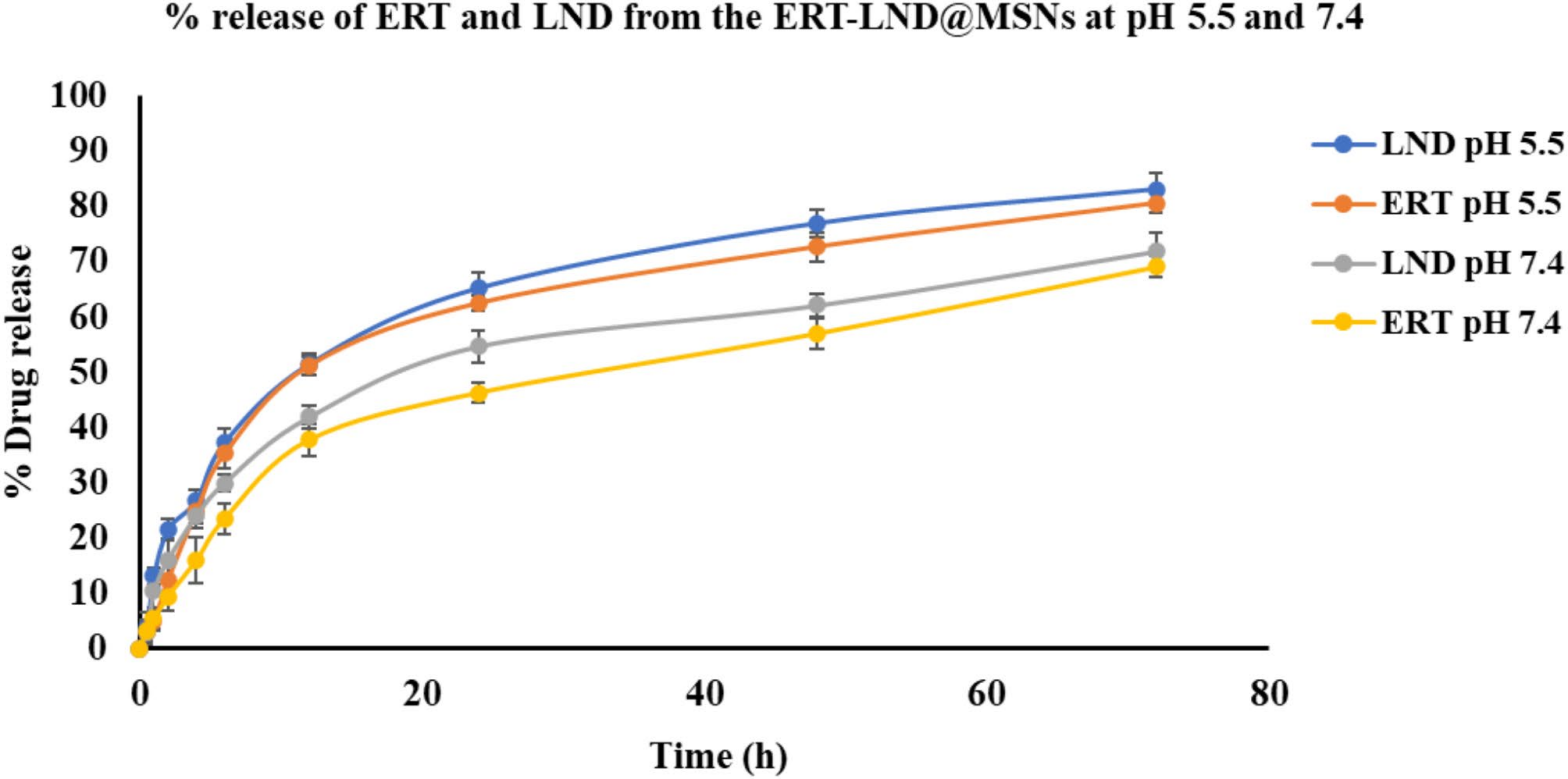
An illustration of % drug release of LND and ERT from the ERT-LND@MSNs at pH 5.5 and pH 7.4.

The developed method was used to evaluate the % drug release of ERT and LND from the ERT-LND@MSNs at pH 5.5 and 7.4. Samples were collected at the following time points: 0, 0.5, 1, 2, 4, 6, 12, 24, 48, and 72 h. The results showed that the ERT-LND@MSNs were stable at both pH levels tested. Both drugs i.e. ERT and LND were released maximum by 72 h at pH 5.5 and pH 7.4. The ERT-LND@MSNs have showed the sustained release of ERT and LND, as it is depicted in Fig. 9. The % release obtained from the ERT-LND@MSNs was found to be 80.59 ± 1.86% for ERT and 83.13 ± 3.16% for LND at pH 5.5, while at pH of 7.4 they were 69.05 ± 1.96% of ERT and 71.88 ± 3.27% of LND.

### Scanning electron microscopy

**Fig. 10:**
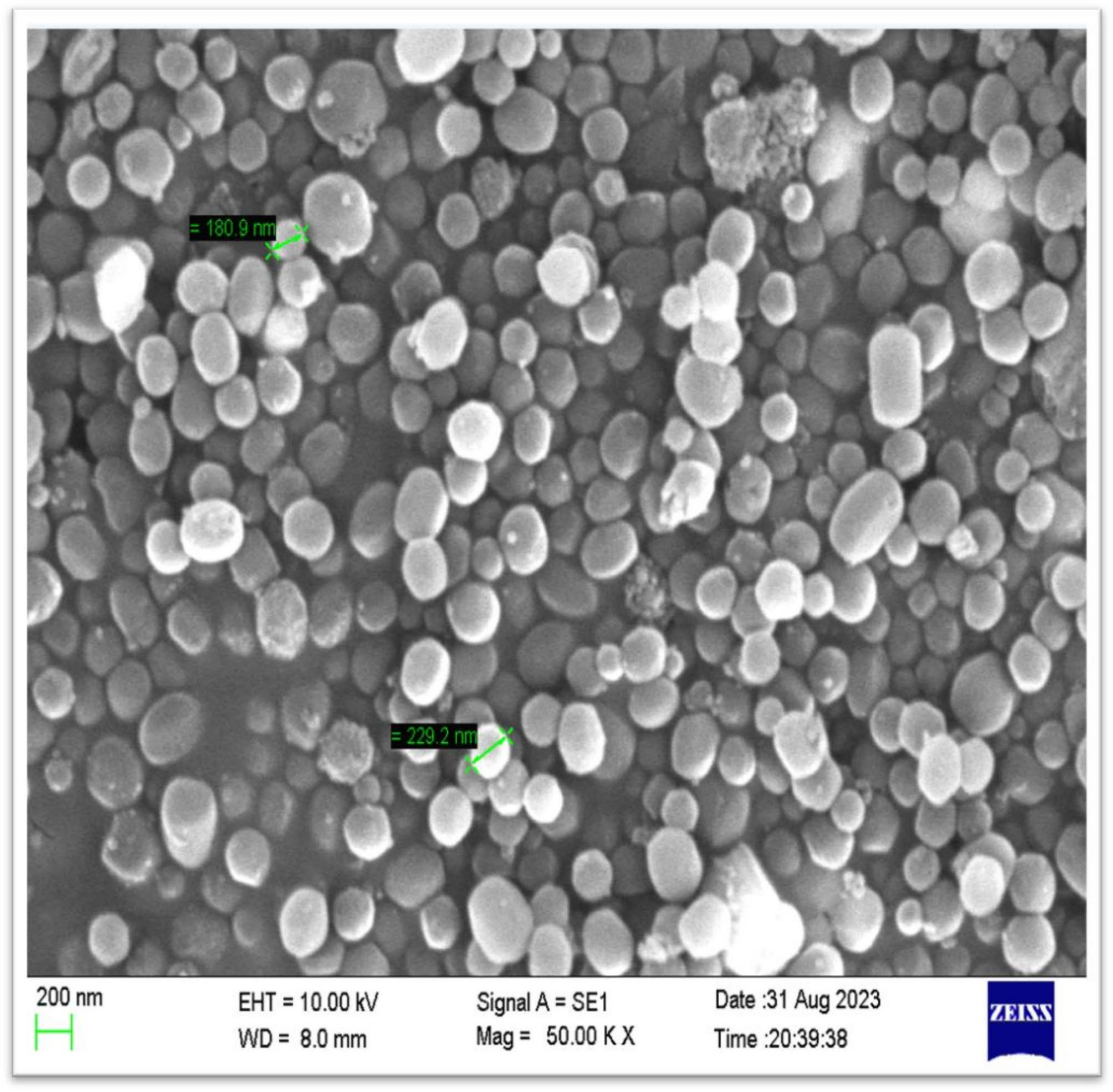
SEM result of ERT and LND loaded MSNs showing average particle size.

The formulation shape and individual particle nanostructure were visible in the ERT@MSNs SEM image (Fig. 10). Changes in the concentrations of TEOS and CTAB can alter the shape of the produced MSNs. When the CTAB and TEOS concentrations in MSNs were adjusted, the average size was determined to be 229.2 nm, and the surface shape was spherical and aggregated.

### Greenness evaluation of the developed HPLC method

**Fig. 11:**
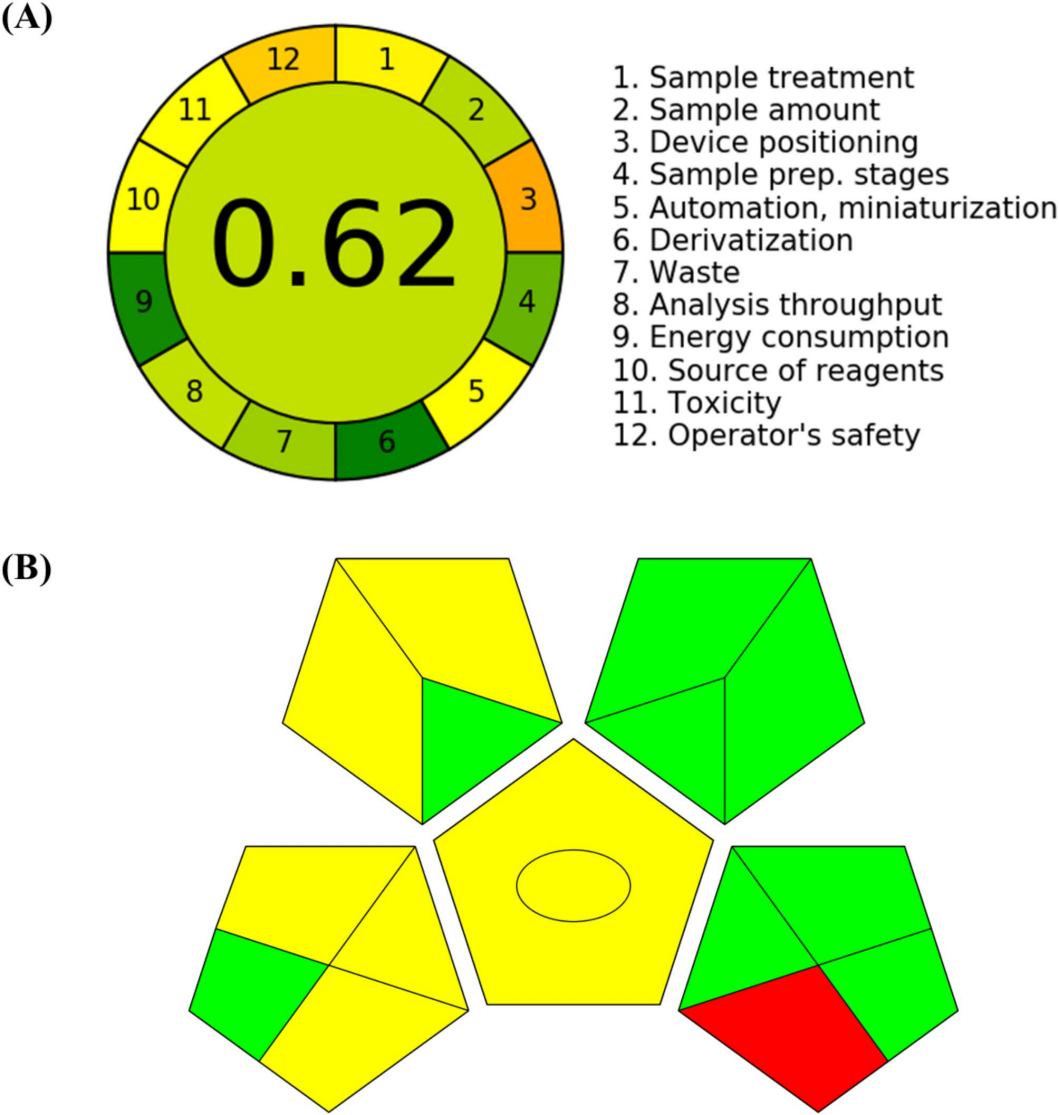
Pictorial representation for greenness profile assessment of RP-HPLC method (**A**) AGREE analysis, (**B**) GAPI analysis.

Evaluating the effects of analytical procedures on the environment, operator safety, and health is the aim of green analytical chemistry (GAC), especially when those techniques require the use of organic solvents in the mobile phase. In recent years, there has been a tremendous increase in interest in GAC, making it feasible to rate the greenness of various analytical approaches using a range of green evaluation tools. Using the AGREE and GAPI program, we created Fig. 11, illustrating the method’s environmental friendliness. The overall AGREE score of the developed HPLC method was 0.62. The GAPI study showed that 8 factors were green, 6 were yellow and 1 was red, indicating that the developed method was environmentally friendly. The suggested HPLC technique for measuring ERT and LND was determined to be environmentally friendly.

## Conclusion

The environmentally friendly RP-HPLC analytical method for the estimation of ERT and LND from the MSNs was successfully done. The method was optimized using the three-level BBD The DoE analysis revealed a desirability of 0.956. The optimized RP-HPLC method was validated as per ICH Q2 (R1) guidelines. All the validation parameters were come under acceptance criteria. To far, no HPLC analytical method has been reported for simultaneous quantifying ERT and LND from the MSNs. The MSNs excipients did not interfere with the ERT and LND Rt. The developed RP-HPLC method was used to analyze the ERT and LND from the ERT-LND@MSNs. The % EE in MSNs of ERT was found to be 72.65% and for LND was found to be 79.50. % DL in MSNs of ERT was found to be 14% and for LND was found to be respectively. SEM analysis have showed that average size of ERT-LND@MSNs was determined to be 229.2 nm, and the surface shape was spherical and aggregated. The developed method used to evaluate the drug release of the ERT-LND@MSNs. The release studies were conducted at pH 5.5 and 7.4. in conclusion we have developed and optimized RP-HPLC analytical method for the quantification of ERT and LND from its formulations. This developed method could be used to check the % DL, % EE, and release profile. The developed method is eco-friendly, economical, robust, accurate and precise.

## Electronic supplementary material

Below is the link to the electronic supplementary material.

Supplementary Material 1

## Data Availability

The datasets generated during the current study are available from the corresponding author on reasonable request.

## Abbreviations

ERT: Erastin
LND: Lenalidomide
BBD: Box–Behnken design
MSNs: Mesoporous silica nanoparticles
OFAT: One factor at a time
DoE: Design of expert
ANOVA: Analysis of variance
ICH: International conference on harmonization
