# Supplementary material for: Quality by design based ecofriendly HPLC analytical method for simultaneous quantification of erastin and lenalidomide in mesoporous silica nanoparticles
Source: Sci Rep. 2025 Mar 14;15:8873. doi: 10.1038/s41598-025-93331-8 (PMC11909239; doi:10.1038/s41598-025-93331-8)
Supplement: Supplementary file 1 — Supplementary Material 1 [file 41598_2025_93331_MOESM1_ESM.docx]

**Supplementary file Data**

**Absorbance maxima for estimation of erastin and lenalidomide**

To determine the LND and ERT wavelengths for the study. Separate preparation of the ERT and LND working solution was done at a concentration of 10 µg/mL. After preparing the final solutions, a double-beam UV-Vis spectrophotometer (UV-1800) operating in the 190–800 nm range was used to estimate its absorption maxima (λmax). The blank in this case was methanol.

**System suitability of the developed HPLC method**

**Table S1: System suitability of the developed HPLC method**

| **Parameters** | **Erastin** | **Lenalidomide** |
| --- | --- | --- |
| **Theoretical plate** | 2794.112667 | 3074.866 |
| **Tailing factor** | 1.494 | 1.141333 |
| **Retention time** | 4.706 | 6.306 |
| **Resolution** | 3.801 | |
| **Specificity** | No interfering peaks at the tR of ERT and LND | |

**Peak purity of the drugs**


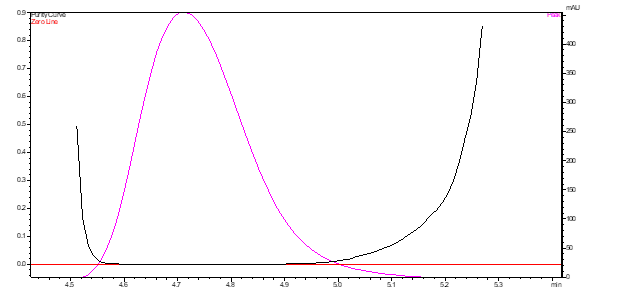


**(A)**


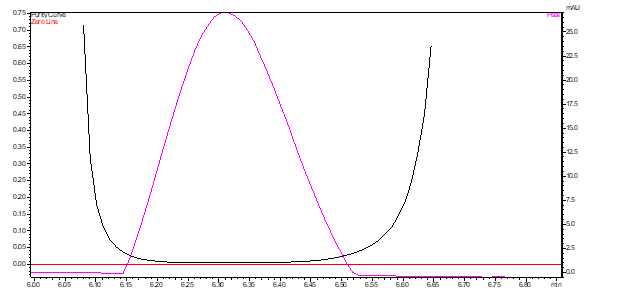


**(B)**

**Fig. S1. Peak purity of the drugs** **(A) Erastin (B) Lenalidomide**

**Calibration curve of erastin and lenalidomide**

**
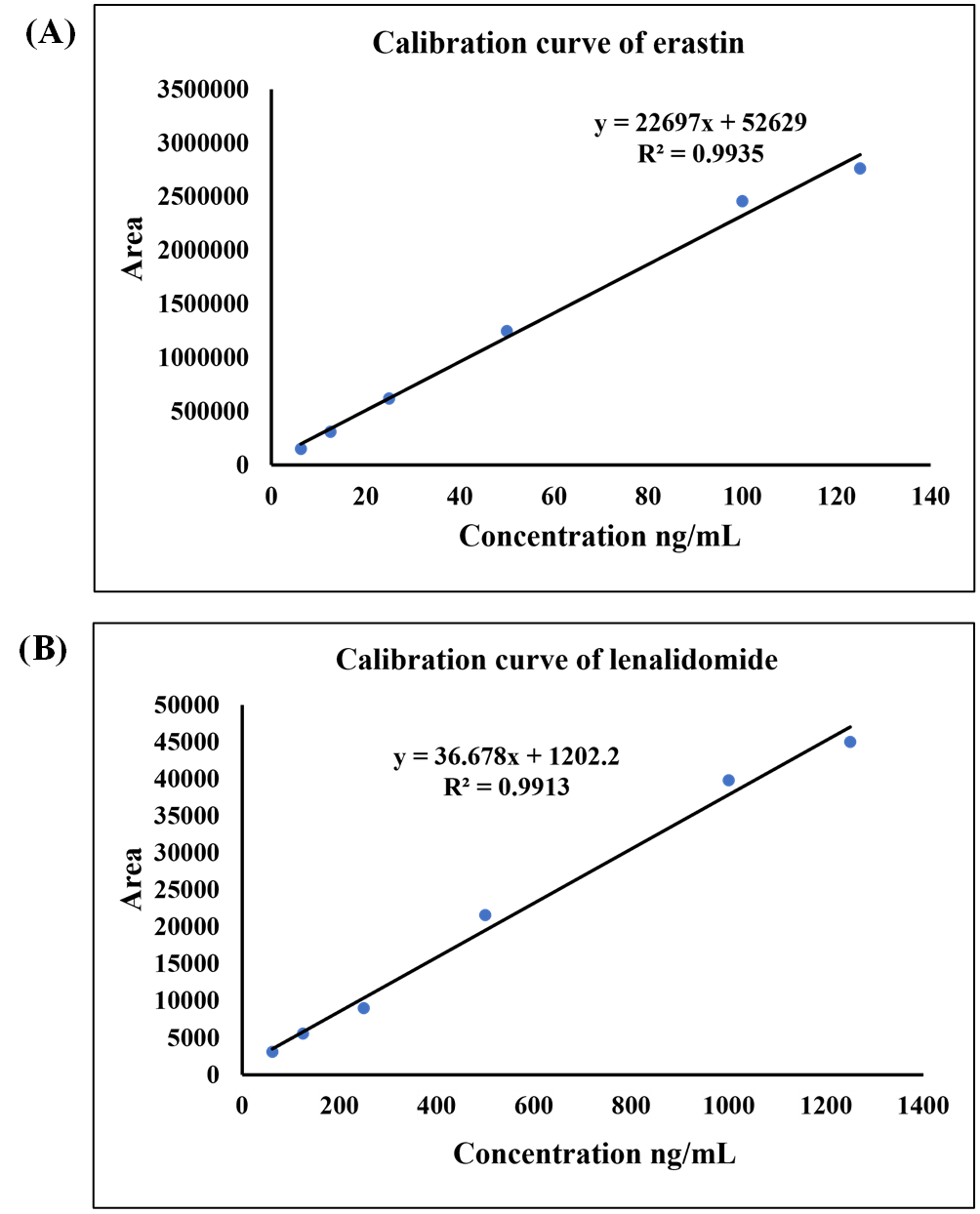
**

**Fig. S2. Calibration curve of the drugs (A) Erastin (B) Lenalidomide**

**Table S2: Results of robustness study**

| **Robustness** | | | | | |
| --- | --- | --- | --- | --- | --- |
| **Parameters** | | **Erastin** | | **Lenalidomide** | |
|  |  | **Rt** | **Tailing** | **Rt** | **Tailing** |
| **pH** | **5.75** | 4.716 ± 0.001 | 1.22 ± 0.01 | 6.316 ± 0.003 | 1.54 ± 0.08 |
|  | **5.85** | 4.724 ± 0.004 | 1.42 ± 0.03 | 6.381 ± 0.002 | 1.45 ± 0.02 |
| **Temperature (℃)** | **24** | 4.768 ± 0.007 | 1.26 ± 0.05 | 6.318 ± 0.002 | 1.26 ± 0.05 |
|  | **26** | 4.714 ± 0.003 | 1.27 ± 0.09 | 6.399 ± 0.008 | 1.34 ± 0.07 |
| **Flow rate (ml/min)** | **0.7** | 4.817 ± 0.002 | 1.52 ± 0.01 | 6.327 ± 0.007 | 1.35 ± 0.07 |
|  | **0.9** | 4.612 ± 0.004 | 1.25 ± 0.03 | 6.492 ± 0.003 | 1.65 ± 0.02 |
| **Injection volume (µl)** | **9** | 4.718 ± 0.004 | 1.15 ± 0.02 | 6.311 ± 0.006 | 1.31 ± 0.03 |
|  | **11** | 4.714 ± 0.003 | 1.54 ± 0.07 | 6.304 ± 0.002 | 1.43 ± 0.07 |
| **Wavelength (nm)** | **1** | 4.726 ± 0.005 | 1.47 ± 0.01 | 6.356 ± 0.002 | 1.54 ± 0.06 |
|  | **-1** | 4.713 ± 0.003 | 1.36 ± 0.08 | 6.551 ± 0.007 | 1.35 ± 0.08 |

**Chromatogram of robustness study of ERT and LND**


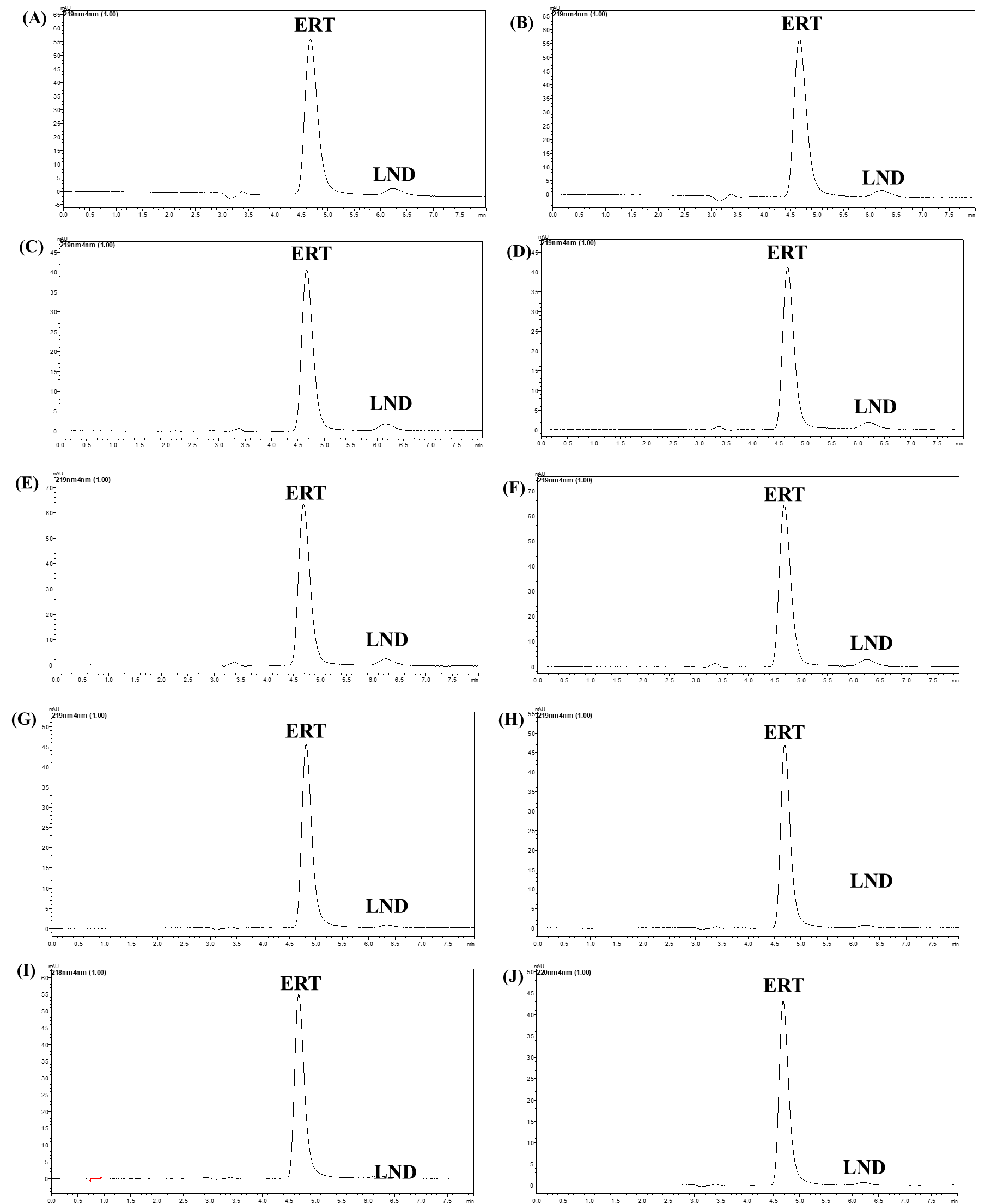


**Fig. S3.** Chromatogram forced degradation of erastin (ERT) and lenalidomide (LND). (A)pH 5.75, (B) pH-5.85, (C) Flow 0.7 mL/min, (D) Flow 0.9 mL/min, (E) Temperature 24℃, (F) Temperature 26℃, (G) Injection volume 9 µL, (H) Injection volume 11 µL, (I) Wavelength 218 nm, (J) Wavelength 220 nm.

**Chromatogram of force degradation study of ERT and LND**

**
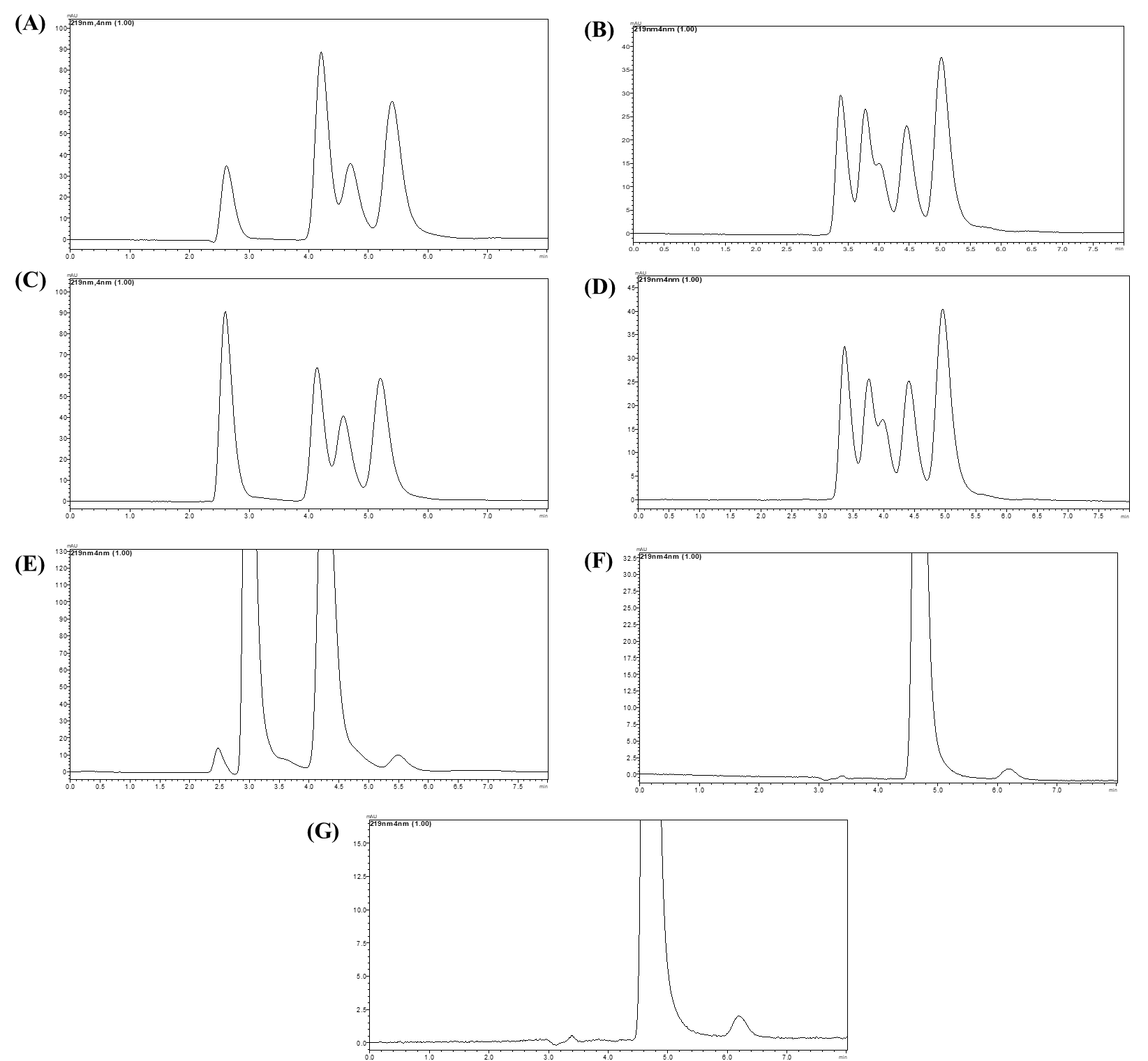
**

**Fig. S4.** Typical chromatogram forced degradation of erastin (ERT) and lenalidomide (LND). (A) Acid-induced hydrolysis by 0.1 N HCl, (B) Acid-induced hydrolysis by 1 N HCl, (C) Base-induced hydrolysis by 0.1 N NaOH, (D) Base-induced hydrolysis by 1 N NaOH, (E) Oxidative stress by 3% H2O2, (F) Thermal stress (60℃), (G) Photothermal stress.
